# The protective effect of housing affordability on childhood asthma risk: a longitudinal fixed-effects analysis

**DOI:** 10.1093/aje/kwag013

**Published:** 2026-01-20

**Authors:** Yuxi Li, Ankur Singh, Rebecca Bentley

**Affiliations:** Centre for Health Policy, School of Population and Global Health, University of Melbourne, Melbourne, Australia; University of Sydney School of Dentistry, University of Sydney, Sydney, Australia; Charles Perkins Centre, University of Sydney, Sydney, NSW, Australia; Centre for Epidemiology and Biostatistics, School of Population and Global Health, University of Melbourne, Melbourne, Australia; Centre for Health Policy, School of Population and Global Health, University of Melbourne, Melbourne, Australia

**Keywords:** housing affordability, rent assistance, asthma, longitudinal, Australia

## Abstract

Childhood asthma is influenced by early-life social conditions, yet few studies have evaluated housing affordability as a modifiable structural exposure. We used data from six biennial waves (2006-2018) of the Longitudinal Study of Australian Children to assess whether changes in housing affordability and rental assistance were associated with incident asthma in childhood. Fixed-effects logistic regression models were used to estimate within-child associations between time-varying housing exposure and asthma outcomes. The main analytic sample included 3773 children asthma-free at baseline; a subsample of 522 children in low-income renting households was used to evaluate rental assistance. Transitions into affordable housing were associated with a 31% reduction in asthma risk (OR, 0.69; 95% CI, 0.52-0.90). Among low-income private renters, new receipt of assistance was associated with 65% lower odds of asthma onset (OR, 0.35; 95% CI, 0.14-0.85). No associations were observed for asthma severity. Sensitivity analyses using lagged exposures, alternative definitions of affordability, and unadjusted income models yielded consistent findings. These findings support housing affordability as a potential policy lever for asthma prevention and demonstrate the utility of within-person designs for strengthening causal inference in observational evaluation of structural interventions.

## Introduction

Childhood asthma is among the most prevalent chronic diseases globally, significantly affecting children's quality of life, healthcare utilization, and long-term health outcomes.^1^ Its burden falls disproportionately on children in socioeconomically disadvantaged households, contributing to persistent health inequities.^2^ A substantial body of research has established links between poor housing conditions—such as dampness, mold, and inadequate ventilation—and asthma onset and severity in children.^3^^,^^4^ These environmental exposures remain central to clinical and public health strategies for asthma prevention.

In recent years, broader structural housing factors have gained attention as potential upstream determinants of child respiratory health.^5^ Among these, housing affordability has emerged as a growing concern. Housing affordability stress (HAS) refers to the economic strain experienced by households when a large proportion of income is spent on housing, often leaving insufficient resources for other essential needs such as healthcare, food, and transportation.^6^ In Australia and in many other high-income countries, affordability stress is commonly measured using the 30/40 rule; that is, households in the lowest 40% of the income distribution that spend more than 30% of their income on housing are considered to be in affordability stress.^7^ This threshold is widely used in housing policy to identify families at risk of material hardship.^8^

For children, living in unaffordable housing may increase asthma risk through multiple pathways, including reduced housing quality (increasing exposure to allergens, cold temperatures and/or damp and mold), frequent relocation, and family stress.^9^ Families burdened by high housing costs are more likely to reside in substandard conditions with poor indoor air quality, a known risk factor for respiratory conditions.^2^ Additionally, unaffordable housing can disrupt medical care and asthma management through housing instability and financial trade-offs that increase psychosocial stress.^10^^,^^11^

Despite these plausible pathways, the relationship between housing affordability and childhood asthma has received limited direct attention in empirical research. Most existing studies on housing and asthma focus on dwelling conditions or neighborhood environments, rather than affordability as a distinct and modifiable exposure.^12^ Where affordability has been examined in the broader health literature, studies often rely on cross-sectional or between-group comparisons, which limit causal interpretation and fail to account for how changes in affordability over time may shape child health trajectories.^13-15^

What remains unclear—and is rarely examined—is whether improvements in housing affordability might protect against asthma onset. This is an important area of focus because affordability can be modified through financial assistance measures in a short timeframe. This study adopts a novel analytic perspective by reframing affordability as a potentially asthma-protective intervention because it supports housing security, condition and reduces financial stress. Specifically, we seek to examine whether housing stress relief is associated with reduced asthma risk.

In parallel, many jurisdictions operate demand-side rent assistance to lower rent burdens for low-income renters. In Australia, the principal program is Commonwealth Rent Assistance (CRA), a fortnightly, non-taxable cash supplement for low-income private renters when rent exceeds program thresholds up to a capped, twice-yearly indexed maximum.^16^ Thresholds and maximum rates are set under national social security policy and are administered by Services Australia (see Appendix S1 for administrative details). Commonwealth Rent Assistance primarily reduces the rent-to-income ratio. Comparable demand-side programs include the United Kingdom's Housing Benefit^17^ or Universal Credit housing element,^18^ New Zealand’s Income-Related Rent Subsidy,^19^ and Canada’s Housing Benefit.^20^ In some settings, such assistance have been linked to improvements in housing stability and quality, and reductions in asthma triggers in the home such as mold, pests, and overcrowding (associated with exposure to viral infections).^21^^,^^22^ Yet their direct impact on childhood asthma prevention through a reduction in incident asthma or severity of asthma symptoms has not been evaluated.^2^ Because randomized trials are often infeasible, well-designed longitudinal studies with rigorous analytic strategies are needed to inform policy. Accordingly, we use 14 years of longitudinal data collected on Australian children and fixed effect regression models to examine the potentially protective effects of housing policy that support affordable housing. Specifically:

(1) Does improved housing affordability reduce the risk of asthma onset in childhood?
(2) Among low-income private renters, does receipt of rental assistance reduce the risk of asthma onset?

A secondary objective was to explore whether these housing exposures are also associated with asthma severity among children with asthma. Although this analysis was considered exploratory, it aimed to assess whether improvements in affordability might mitigate symptom burden or reduce acute disease indicators.

By applying fixed effects (FE) models to estimate within-child changes over time, this study isolates the effect of changing housing circumstances on asthma risk, controlling for all time-invariant child and family characteristics. The findings contribute new evidence on the potential of structural housing interventions to reduce asthma incidence and promote respiratory health equity in early life.

## Methods

### Study design and analytical framework

This study used data from the Longitudinal Study of Australian Children (LSAC), a nationally representative population-based cohort that has followed two cohorts of Australian children since 2004. The LSAC collects rich, repeated measures on child health, housing, family circumstances, and socioeconomic conditions, making it well suited to examine how structural conditions shape child health over time.^23^

This analysis used waves 2 (age 2 years) to 7 (age 14 years). Wave 1 was excluded because parent-reported asthma diagnoses were not yet collected. The aim is to examine whether changes in housing affordability—specifically, transitioning into affordable housing or beginning to receive rental assistance—were associated with changes in asthma incidence or severity.

Fixed effects regression models were applied to estimate these associations. This approach compares each child to themselves over time, effectively controlling for all time invariant characteristics including unmeasured factors such as genetic predisposition, family background, and baseline health status.^24^ The fixed-effects coefficient estimates the within-child contrast of being in affordable versus unaffordable housing. Children contribute information whenever their exposure changes in either direction across waves. Rather than asking whether children in affordable housing have lower asthma risk than those in unaffordable housing, this approach asks: *When a given child’s housing becomes more affordable, do their asthma outcomes improve compared to periods when housing was unaffordable?* Similarly, it asks: *Does a child’s asthma risk change in the period when their family begins to receive rental assistance, compared to periods when they did not?*

### Study sample

Children were eligible for inclusion if they (1) participated in wave 2 of LSAC (study baseline), (2) had no parent-reported asthma at baseline, and (3) contributed at least two waves of asthma outcome data between waves 2 and 7.

Of the 4606 children who participated in wave 2628 were excluded due to having been diagnosed already with asthma at baseline, and 204 were excluded for contributing fewer than two waves of asthma outcome data. This resulted in an eligible study sample of 3773 children.

For analyses of housing affordability, FE regression models were estimated using all eligible children. As the FE approach relies on within-person change, only individuals with variation in both the exposure and the outcome contributed information to model estimation. Final model-specific sample sizes are reported with the regression results.

**Figure 1:**
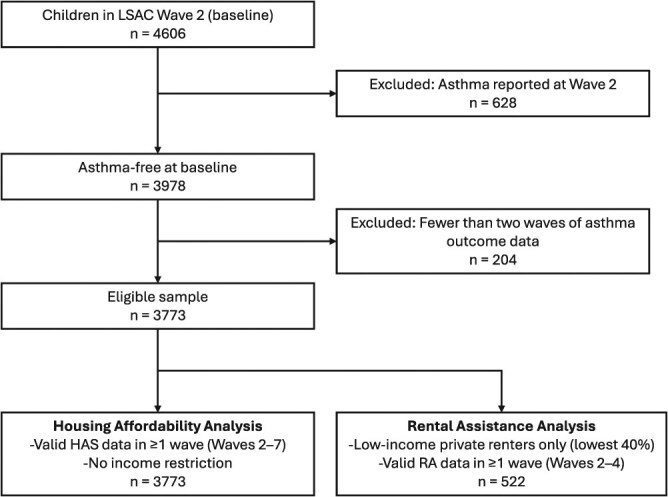
STROBE diagram of sample derivation for housing affordability and rental assistance analyses. Children with parent-reported asthma at wave 2 were excluded to define a baseline asthma-free population. An additional 50 children with fewer than two waves of asthma outcome data were removed. All 3928 eligible children were included in the housing affordability analysis. Rental assistance models were restricted to low-income children (lowest 40% of income distribution) in private renting households with valid data in at least one wave.

For analyses of rental assistance, we restricted to a subsample of low income private renters (bottom 40% of the equivalized national income distribution), yielding 522 children. The sample flow is shown in the STROBE diagram (Figure 1).

### Exposure variables

Two time-varying housing exposures were examined: (1) transitions into affordable housing, and (2) initiation of CRA receipt.

Housing affordability stress was measured using the standard 30/40 rule, a widely accepted benchmark in housing policy research and practice in Australia^25^ and internationally.^26^ At each wave, households in the lowest 40% of the national equivalized income distribution were classified as unaffordable if more than 30% of their income was spent on housing costs. A binary indicator was constructed for each wave (1, affordable housing; 0, unaffordable housing). Transitions into affordable housing were defined as a change in the affordability indicator from 0 to 1 across waves.

Commonwealth Rent Assistance is parent-reported in LSAC and available in waves 2 to 4. We coded receipt as a time-varying binary indicator each wave (1 = receiving, 0 = not) and conducted CRA analyses in the low-income private-renter subsample. Initiation was defined as a change from 0 to 1 between waves.

### Outcome variables

The primary outcome was incident asthma, defined as a new parent-reported diagnosis between waves 2 and 7. At each wave, caregivers were asked, “Has a doctor ever told you that your child has asthma?” Children were considered to have developed incident asthma if their response changed from “No” to “Yes” during follow-up.

A secondary outcome, asthma severity, was assessed among children with asthma and based on two caregiver-reported indicators: (1) asthma medication use in the past 12 months and (2) hospitalization for asthma during the same period. These indicators were combined into a binary variable: moderate or severe asthma (defined as medication use and/or hospitalization) versus mild asthma (diagnosis only). This approach is consistent with population-based classifications, where medication use and hospital admissions serve as proxies for underlying disease activity and exacerbation risk.^27^ An ordinal severity variable (mild, moderate, severe) was explored in preliminary models but not used in final analyses due to limited within-child variation and poor model convergence. However, the full ordinal distribution is reported descriptively to provide a granular view of asthma burden over time.

### Covariates

Time-varying covariates were selected *a priori* based on a directed acyclic graph (Figure S1) and knowledge of potential confounding pathways. All models included equivalized household income (OECD-modified equivalence scale: 1.0 first adult, 0.5 additional adults aged ≥15 years, 0.3 per child <15 years^28^) centered at the wave-specific sample mean and scaled in $100 increments, household size, interviewer-rated dwelling condition, and area-level socioeconomic disadvantage, measured using the Socio-Economic Indexes for Areas (SEIFA) Index of Relative Socioeconomic Disadvantage.^29^

The fixed-effect approach inherently adjusts for all time-invariant characteristics, whether measured or unmeasured.

### Statistical analysis

Descriptive analyses summarized baseline characteristics and examined temporal trends in asthma outcomes. Wave-to-wave transition probabilities were calculated to describe the dynamics of transitioning into or out of affordable housing. The distribution of exposure duration, defined as the number of waves a child lived in affordable housing, was also examined to contextualize exposure persistence.

Child-level fixed-effects logistic regression was used to estimate within-child associations between exposures and outcomes. Separate models were fitted for each exposure–outcome pair. Incident asthma was analyzed among children without asthma at baseline; asthma severity (moderate/severe vs. mild) was modeled among children with asthma. Commonwealth Rent Assistance–related analyses were restricted to low-income private renters.

Patterns of missingness were examined across key exposure, outcome, and covariate variables. The proportion of missing data was low for most variables: < 1% for SEIFA quintile and household size, 2% for dwelling condition, 6% for housing affordability, and < 1% for asthma outcomes. Analyses were conducted using complete case estimation in Stata 18 (StataCorp, College Station, TX).

### Sensitivity analyses

Three analyses were specified as *a priori* to assess the sensitivity of findings to alternative operationalizations of exposure, temporal assumptions, model covariate structures, and unmeasured confounding. First, an alternative definition of housing affordability was applied using the conventional 30% rule, which classifies households as unaffordable if they spend 30% or more of gross income on rent, regardless of income level. This measure is widely used in housing research and policy internationally, particularly in North America and Europe, and remains a dominant benchmark in the empirical literature despite its known limitations for capturing affordability burden among low-income households.^30^^,^^31^ Second, models were re-estimated using a one-wave lag between exposure and outcome, assigning housing affordability status in wave t to predict asthma outcomes in wave t + 1, to account for temporal ordering and potential effect latency. Finally, *E*-values was calculated to quantify the minimum strength of association that an unmeasured confounder would need to have with both the exposure and the outcome to fully explain away the observed association.^32^

## Results

Among the 3773 children without asthma at baseline (wave 2), 13.1% (*n* = 496) were classified as living in HAS (Table 1). Children in the HAS group were more likely to live in single-parent households (30.4% vs 5.4%), reside in dwellings rated as fair or poor condition (36.3% vs. 21.6%), and live in socioeconomically disadvantaged areas, with 53.8% in the lowest two SEIFA quintiles compared to 40.8% of the non-HAS group. Average equivalized weekly household income was substantially lower in the HAS group (AUD $305; SD, 105) than in the affordable housing group (AUD $822; SD, 480). Educational disadvantage was also more prevalent; 19.3% of mothers in the HAS group had not completed Year 12, compared to 12.1% in the non-HAS group. There were no notable differences in gender or household size.

**Table 1:** Baseline (wave 2) characteristics of children without asthma, by HAS status.^a^

**Characteristics, *n* (%)**	**Overall (*N* = 3773)**	**Unaffordable (*n* = 496)**	**Affordable (*n* = 3083)**
Sex			
Male	1881 (49.9)	248 (50.0)	1534 (49.8)
Female	1892 (50.1)	248 (50.0)	1549 (50.2)
Equivalized household weekly income (AUSD), mean (SD)	727.6 (479.8)	304.7 (104.7)	822.1 (479.5)
Tenure			
Owned with mortgage	2337 (61.9)	251 (50.6)	2047 (66.4)
Owned outright	373 (9.9)	16 (3.2)	211 (6.8)
Renting	953 (25.3)	229 (46.2)	718 (23.3)
Others	110 (2.9)	0 (0.0)	107 (3.5)
Single parent household	346 (9.2)	151 (30.4)	167 (5.4)
Dwelling condition			
Badly deteriorated	8 (0.2)	1 (0.2)	5 (0.2)
Poor condition	95 (2.5)	19 (3.8)	74 (2.4)
Fair condition	750 (19.9)	148 (29.8)	559 (18.1)
Good condition	2865 (75.9)	321 (64.7)	2402 (77.9)
Missing	55 (1.5)	7 (1.4)	43 (1.4)
No. of people in household, mean (SD)	4.3 (1.1)	4.4 (1.5)	4.3 (1.1)
SEIFA quintile^b^			
Most disadvantaged	764 (20.2)	120 (24.2)	574 (18.6)
Disadvantaged	835 (22.1)	136 (27.4)	659 (21.4)
Middle	884 (23.4)	111 (22.4)	729 (23.6)
Advantaged	674 (17.9)	75 (15.1)	582 (18.9)
Most advantaged	616 (16.3)	54 (10.9)	539 (17.5)
Maternal education			
Year 8 or below	31 (0.8)	11 (2.2)	18 (0.6)
Year 9 to 11	469 (12.4)	85 (17.1)	355 (11.5)
Year 12 or certificate	1910 (50.6)	308 (62.1)	1493 (48.4)
Bachelor or above	1353 (35.9)	91 (18.3)	1208 (39.2)
missing	10 (0.3)	1 (0.2)	9 (0.3)

Abbreviations: AUSD, Australian dollars; HAS, housing affordability stress; SEIFA, Socio-Economic Indexes for Areas.

^a^HAS was missing for 194 children (5.1%) at baseline. These children are included in the overall descriptive sample but not shown in the affordability-stratified columns.

^b^SEIFA quintiles reflect area-level socioeconomic disadvantage, based on the Index of Relative Socioeconomic Disadvantage.

In the low-income private renter subsample at baseline (wave 2), 577 households were included, of which 235 (40.7%) reported CRA receipt. Compared with non-recipients, recipients were more often in single-parent households (48.1% vs 36.8%) and in the bottom income quintile (67.7% vs 57.4%); other characteristics were similar (Table S1). Across waves 2 to 4, CRA receipt was reported in 675 of 1675 child-waves (40.3%), with proportions stable over time (≈40%).

**Figure 2:**
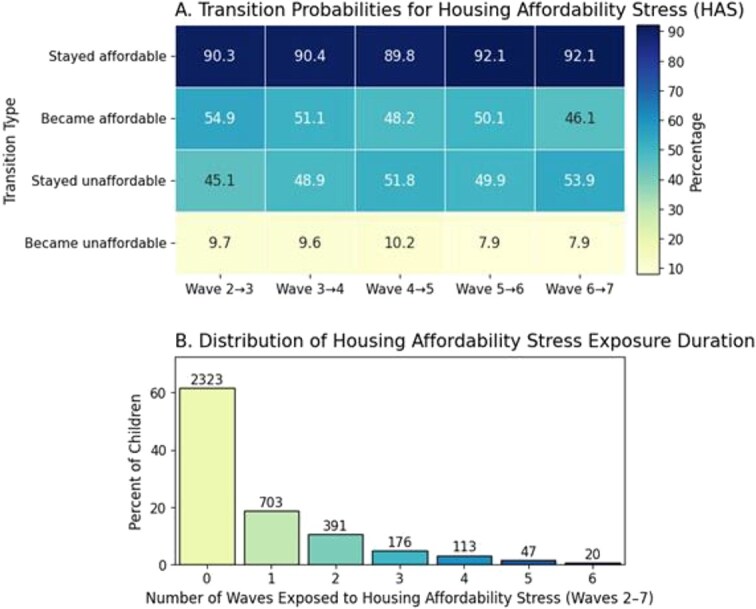
Transitions and duration of HAS, waves 2-7. Panel a. wave-to-wave transition probabilities for HAS between consecutive survey waves. Panel B. Distribution of cumulative exposure to HAS across follow-up (number of waves exposed, waves 2-7). Percentages are based on children with non-missing HAS data at each wave (*n* = 3773). A line-plot version of panel (A) is available in the supplement (Figure S2). Abbreviation: HAS, housing affordability stress.

Figure 2 presents wave-to-wave transition probabilities in HAS status. Most children in affordable housing remained so at the next wave (90-92%). In contrast, HAS was more transient: between 46% and 55% of children living in unaffordable housing transitioned into affordability in the following wave. Transitions from affordable to unaffordable housing were less common (7.9-10.2%) but consistently observed. Regarding exposure duration, 70% of child-wave observations were unexposed to HAS, while 16% involved two or more episodes of HAS exposure, and 3% reflected chronic exposure across five or more waves. These patterns indicate meaningful within-child variation in affordability status over time, supporting the use of longitudinal FE models.

**Figure 3:**
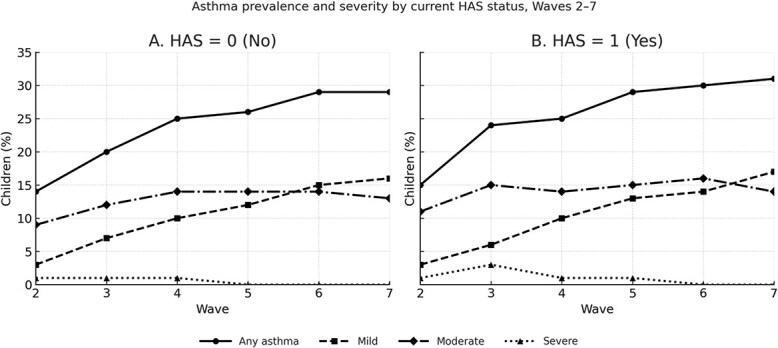
Asthma prevalence and severity by current HAS, waves 2-7Panel a shows children in current affordable housing (HAS = 0); panel B shows those in current unaffordable housing (HAS = 1). Lines indicate the percentage of children with any asthma and, separately, mild, moderate, or severe asthma at each survey wave. Abbreviation: HAS, housing affordability stress.

Asthma prevalence increased with age, from 13.7% at wave 2 to 29.1% at wave 7. As shown in Figure 3, children currently living in unaffordable housing (HAS, yes) had higher asthma prevalence at each wave compared with those in affordable housing. By wave 7, 32.3% of children in the HAS group had developed asthma, compared to 26.8% among those not exposed. Moderate asthma was more common among HAS-exposed children: 11.2% vs. 6.9% by wave 7. Severe asthma remained rare in both groups but was slightly more prevalent among children exposed to HAS during early waves.

Fixed effects regression results are presented in Table 2. Transitions into affordable housing were associated with lower odds of incident asthma (adjusted odds ratio [aOR], 0.69; 95% CI, 0.52-0.90). No association was observed between affordability transitions and asthma severity (aOR, 0.88; 95% CI, 0.55-1.41). Among children in low-income private-renting households, receipt of rental assistance was also associated with reduced asthma incidence (aOR, 0.35; 95% CI, 0.14-0.85), though estimates were based on a small sample (*n* = 155). Rental assistance was not associated with asthma severity (aOR, 0.21; 95% CI, 0.01-4.89).

**Table 2:** Fixed effects logistic regression results for the association between housing exposures and childhood asthma outcomes.

**Exposure**	**Outcome**	**Child-wave observation**	**No. of children**	**Adjusted OR (95% CI)**
**Main analyses**				
Affordable housing	Incident asthma	3468	678	0.69 (0.52–0.90)
Rental assistance^a^	Incident asthma	402	155	0.35 (0.14–0.85)
Affordable housing	Moderate/severe vs. mild asthma	1058	279	0.88 (0.55–1.41)
Rental assistance^a^	Moderate/severe vs. mild asthma	40	20	0.21 (0.01–4.89)
**Sensitivity analyses**				
30% rule (alt. definition)	Incident asthma	3468	678	0.68 (0.56–0.83)
No income adjustment	Incident asthma	3468	678	0.74 (0.57–0.96)
Lagged model^b^	Incident asthma	3481	674	0.72 (0.54–0.95)

Abbreviations: CI, confidence interval; OR, odds ratio.

All models adjusted for time-varying household size, interviewer-rated dwelling condition, area-level socioeconomic disadvantage (SEIFA quintile), and equivalized household income (except where noted).

Incident asthma models include only children without asthma at baseline. Severity models include only children with asthma and compare moderate/severe cases (based on medication use and/or hospitalization) to mild cases. Estimates are based only on individuals with within-person change in both exposure and outcome.

^a^Rental assistance analyses were restricted to children living in low-income rental households.

^b^Lagged model assigns exposure at wave t to predict asthma outcome at wave *t* + 1.

Sensitivity analyses supported the robustness of findings. Using the 30% rule to define affordability yielded a similar association with asthma incidence (aOR, 0.68; 95% CI, 0.56-0.83). Excluding income adjustment slightly attenuated the effect (aOR, 0.74; 95% CI, 0.57-0.96). In lagged models assigning affordability exposure at time t to predict asthma incidence at time t + 1, the protective association remained statistically significant (aOR, 0.72; 95% CI, 0.54-0.95). To assess potential unmeasured confounding, *E*-values was calculated. The E-value for the observed association between transitions into affordable housing and asthma incidence (OR, 0.69) was 2.26, and the *E*-value for the upper confidence bound (0.90) was 1.46, indicating that an unmeasured confounder would need to be associated with both the exposure and the outcome by a risk ratio of ≥2.26 to fully explain away the observed effect.

## Discussion

This study provides new longitudinal evidence that improved housing affordability and receipt of rental assistance are associated with a reduced risk of childhood asthma onset. In a nationally representative cohort followed over 12 years, transitions into affordable housing were associated with 31% lower odds of incident asthma, while low-income children whose families began receiving rental assistance experienced a 65% reduction in asthma risk. These associations were robust across multiple sensitivity checks, including alternative definitions of affordability and lagged temporal models.

These findings make two distinct contributions. First, they strengthen the case for housing affordability and assistance as a structural determinant of respiratory health. A recent US study by Pollack et al.^9^ found that participation in a housing mobility program was associated with a 33% reduction in asthma exacerbations (RR, 0.67; 95% CI, 0.53-0.86) and fewer acute care visits. Similarly, Boudreaux et al. (2020) reported that children living in federal rental assistance programs had 32% lower odds of asthma-related emergency department use compared with children on waitlists (OR, 0.68; 95% CI, 0.48-0.96).^33^ Whereas these studies focused on asthma management among already-diagnosed children, our study extends the evidence base to asthma incidence using population-based data and a longitudinal within-child design. This approach reduces confounding by unmeasured stable characteristics and addresses a key gap in the literature: whether structural improvements in housing affordability can influence the onset of disease.

The conceptual contribution of this study lies in reframing housing affordability not simply as a socioeconomic exposure or risk marker, but as a potentially modifiable protective factor. Rather than comparing children in affordable versus unaffordable housing at a single point in time, we examined whether changes in affordability status were associated with changes in health risk within the same child. The consistency of associations across multiple analytic specifications reinforces the plausibility of this temporal relationship. Importantly, the magnitude of the association was not limited to severe or sustained affordability stress: even relatively short-term improvements in affordability were associated with meaningful reductions in asthma risk. This reframing carries implications for policy design: if affordability can be improved—whether through income support, rent regulation, or targeted assistance—there may be measurable gains in asthma prevention. Although housing interventions are often justified on economic or social grounds, these findings suggest a complementary rationale grounded in public health and disease prevention.

Asthma severity was not significantly associated with changes in housing affordability or rental assistance exposure. This may reflect limited statistical power in the subgroup of children with asthma, particularly in the CRA analyses, as well as measurement limitations. The severity indicator (based on medication use and hospitalization) may not capture the full spectrum of symptom burden or day-to-day disease management. Alternatively, housing affordability may exert a greater influence on asthma development than on its clinical progression, particularly in contexts such as Australia where access to medications and primary care is relatively equitable.

Several limitations should be noted. First, asthma outcomes were parent-reported and not clinically confirmed, and the severity proxy (any medication and/or hospitalization) lacks drug class/dose detail and may miss gradients in control. Exposure to HAS was also derived from self-reported income and housing costs, such misclassification is likely non-differential but could bias estimates toward the null. Although the FE design strengthens internal validity by controlling for all time-invariant confounding, it does not account for time-varying factors (eg, income shocks) that may co-occur with changes in housing circumstances. Additionally, because data on rental assistance were limited to the early waves, we were unable to examine long-term effects or clarify mechanisms such as changes in housing quality, residential stability, or neighborhood conditions. These limitations restrict interpretability of causal pathways. With respect to external validity, findings may not generalize to populations underrepresented in the sample, including Aboriginal and Torres Strait Islander children or recent migrants. Differences in rental assistance structures between countries (eg, Australia's cash-based CRA vs. the housing voucher systems in the U.S.) also limit cross-context transportability. Finally, fixed-effects estimates pertain to children who experienced within-person changes in exposure and outcome, the results may not represent the experiences of children with persistently stable or chronically disadvantaged housing conditions.

Despite these limitations, the study offers new insight into the potential for structural housing interventions to influence asthma prevention. The magnitude of the observed association is comparable to several well-established risk factors for childhood asthma. For example second hand smoke, parental stress, and residential dampness.^4^ Given the increasing prevalence of HAS and the high disease burden of asthma, even modest risk reductions associated with housing interventions could yield meaningful population health gains. These results support recent calls to consider housing policy not only as social infrastructure but also as a domain of public health investment^33^^.^

In conclusion, relief from unaffordable housing and receipt of rental assistance were associated with reduced incidence of asthma in Australian children. By reframing affordability as a modifiable protective exposure and applying a within-child analytic design, this study highlights the potential for housing policy to serve as a tool for chronic disease prevention in early life. As affordability pressures intensify across high-income countries, housing interventions should be considered part of a broader public health strategy to improve child health and reduce long-term inequities.

### Supplementary material

Supplementary material is available at the *American Journal of Epidemiology* online.

## Supplementary Material

Web_Material_kwag013

## Data Availability

Data from the Longitudinal Study of Australian Children (LSAC) used in this study were accessed under license via the Australian Data Archive. Requests for access to the LSAC data should be directed to the relevant data custodian.
