# Supplementary material for: The protective effect of housing affordability on childhood asthma risk: a longitudinal fixed-effects analysis
Source: Am J Epidemiol. 2026 Jan 20;195(6):1555–62. doi: 10.1093/aje/kwag013 (PMC13231850; doi:10.1093/aje/kwag013)
Supplement: Web_Material_kwag013 [file web_material_kwag013.docx]

**Supplementary Data**

**Title:** The protective effect of housing affordability on childhood asthma risk: a longitudinal fixed-effects analysis

**Authors**: Yuxi Li, Ankur Singh, Rebecca Bentley

**Contents:**

- Appendix S1. Overview of Australia’s Commonwealth Rent Assistance (CRA)
- Table S1. Baseline characteristics of low-income private renters (Wave 2), by Commonwealth Rent Assistance (CRA) receipt
- Figure S1. Directed acyclic graph (DAG) illustrating hypothesised relationships between housing affordability stress (HAS), incident childhood asthma, and covariates.
- Figure S2. Wave-to-wave transitions in housing affordability stress (HAS), Waves 2–7.

**Appendix S1. Overview of Australia’s Commonwealth Rent Assistance (CRA)**

**Program purpose and design**

Commonwealth Rent Assistance (CRA) is Australia’s main demand-side housing subsidy for low-income private renters. It is a fortnightly, non-taxable payment that reduces rent burden among eligible income-support recipients (1).

**Eligibility**

CRA eligibility is determined through the social security system. Recipients must receive an eligible income-support payment (or Family Tax Benefit Part A above the base rate) and pay rent above a minimum threshold. Tenants of public housing authorities are generally ineligible. Thresholds and maximum rates vary by household composition (1,2).

**Payment calculation**

When reported rent exceeds the relevant threshold, CRA covers 75 cents per additional dollar of rent up to a capped maximum. Thresholds and caps differ by family type and are indexed to the Consumer Price Index (CPI) each year on 20 March and 20 September (3).

**Claim and maintenance process**

CRA is typically assessed automatically when an eligible payment is granted, or when a recipient updates their rent or address details. No separate annual application is required. Services Australia may request proof of rent (e.g., rent certificate or tenancy agreement). Recipients must report changes in rent, address, or travel within 14 days, and may request a review at any time (1,3).

**Why eligibility and receipt may differ in survey data**

- Administrative timing: reporting lags, verification, or review cycles may delay commencement or adjustment (4).
- Payment linkage: starting or ceasing an income-support payment automatically changes CRA status.
- Rent fluctuations: small rent changes near thresholds or caps can move households into or out of eligibility.
- Tenure transitions: moving into public housing generally terminates CRA eligibility.

**Administrative benchmarks**

Program parameters are set under national social security policy and administered by Services Australia. Thresholds, caps, and aggregate recipient data are published regularly by Services Australia and the Australian Institute of Health and Welfare (AIHW).

**Reference**

1. Services Australia. *Rent Assistance: How Much You Can Get.* Canberra (ACT): Services Australia; updated Mar 20 and Sep 20 each year. Accessed October 10, 2025.
2. Department of Social Services. *Social Security Guide: 3.8.1 Rent Assistance—Qualification and Payability.* Canberra (ACT): Australian Government, DSS; updated regularly. Accessed October 10, 2025.
3. Department of Social Services. *Social Security Guide: 5.1.7.10 Rent Assistance—Current Rates and Indexation.* Canberra (ACT): Australian Government, DSS; updated regularly. Accessed October 10, 2025.
4. Australian Institute of Health and Welfare. *Housing Assistance in Australia: Overview and CRA Quarterly Data.* Canberra (ACT): AIHW; updated regularly. Accessed October 10, 2025.

**Table S1. Baseline characteristics of low-income private renters (Wave 2), by Commonwealth Rent Assistance (CRA) receipt**

| Characteristic, n (%) | Overall (n=577) | No CRA  (n=340) | CRA (n=235) |
| --- | --- | --- | --- |
| Sex |  |  |  |
| Male | 270 (46.8) | 160 (47.1) | 110 (46.8) |
| Female | 307 (53.2) | 180 (52.9) | 125 (53.2) |
| Equivalised household weekly income (AUSD), mean (SD) | 325.8 (134.6) | 326.3 (146.8) | 325.7 (115.3) |
| Single parent household | 239 (41.4) | 125 (36.8) | 113 (48.1) |
| Dwelling condition |  |  |  |
| Badly deteriorated | 4 (0.7) | 2 (0.6) | 2 (0.9) |
| Poor condition | 47 (8.1) | 31 (9.1) | 15 (6.4) |
| Fair condition | 218 (37.8) | 127 (37.4) | 91 (38.7) |
| Good condition | 294 (51.0) | 171 (50.3) | 122 (51.9) |
| Missing | 14 (2.4) | 9 (2.6) | 5 (2.1) |
| No. of people in household, mean (SD) | 4.3 (1.5) | 4.4 (1.6) | 4.1 (1.3) |
| SEIFA quintile |  |  |  |
| Most Disadvantaged | 165 (28.6) | 97 (28.5) | 67 (28.5) |
| Disadvantaged | 161 (27.9) | 94 (27.6) | 66 (28.1) |
| Middle | 128 (22.2) | 75 (22.1) | 53 (22.6) |
| Advantaged | 78 (13.5) | 43 (12.6) | 35 (14.9) |
| Most Advantaged | 45 (7.8) | 31 (9.1) | 14 (6.0) |
| Maternal education |  |  |  |
| Year 8 or below | 18 (3.1) | 14 (4.1) | 4 (1.7) |
| Year 9 to 11 | 141 (24.4) | 84 (24.7) | 57 (24.3) |
| Year 12 or certificate | 333 (57.7) | 191 (56.2) | 140 (59.6) |
| Bachelor or above | 84 (14.6) | 50 (14.7) | 34 (14.5) |
| missing | 1 (0.2) | 1 (0.3) | 0 (0.0) |


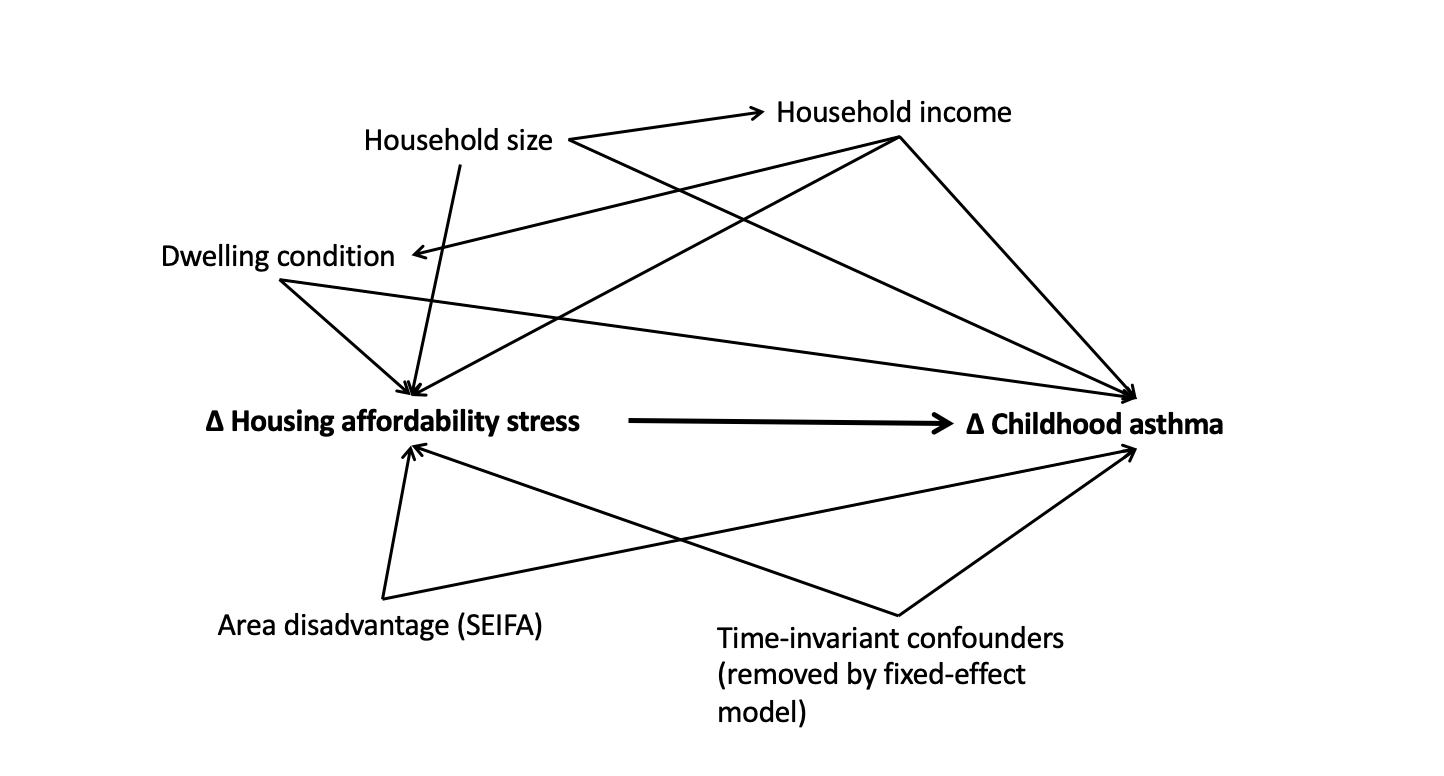
 **Figure S1. Directed acyclic graph (DAG) illustrating hypothesised relationships between housing affordability stress (HAS), incident childhood asthma, and covariates.**

Household income, household size, dwelling condition, and area disadvantage (SEIFA) were adjusted for in all models. Each covariate is assumed to influence both housing affordability and asthma risk through independent socioeconomic and environmental pathways.


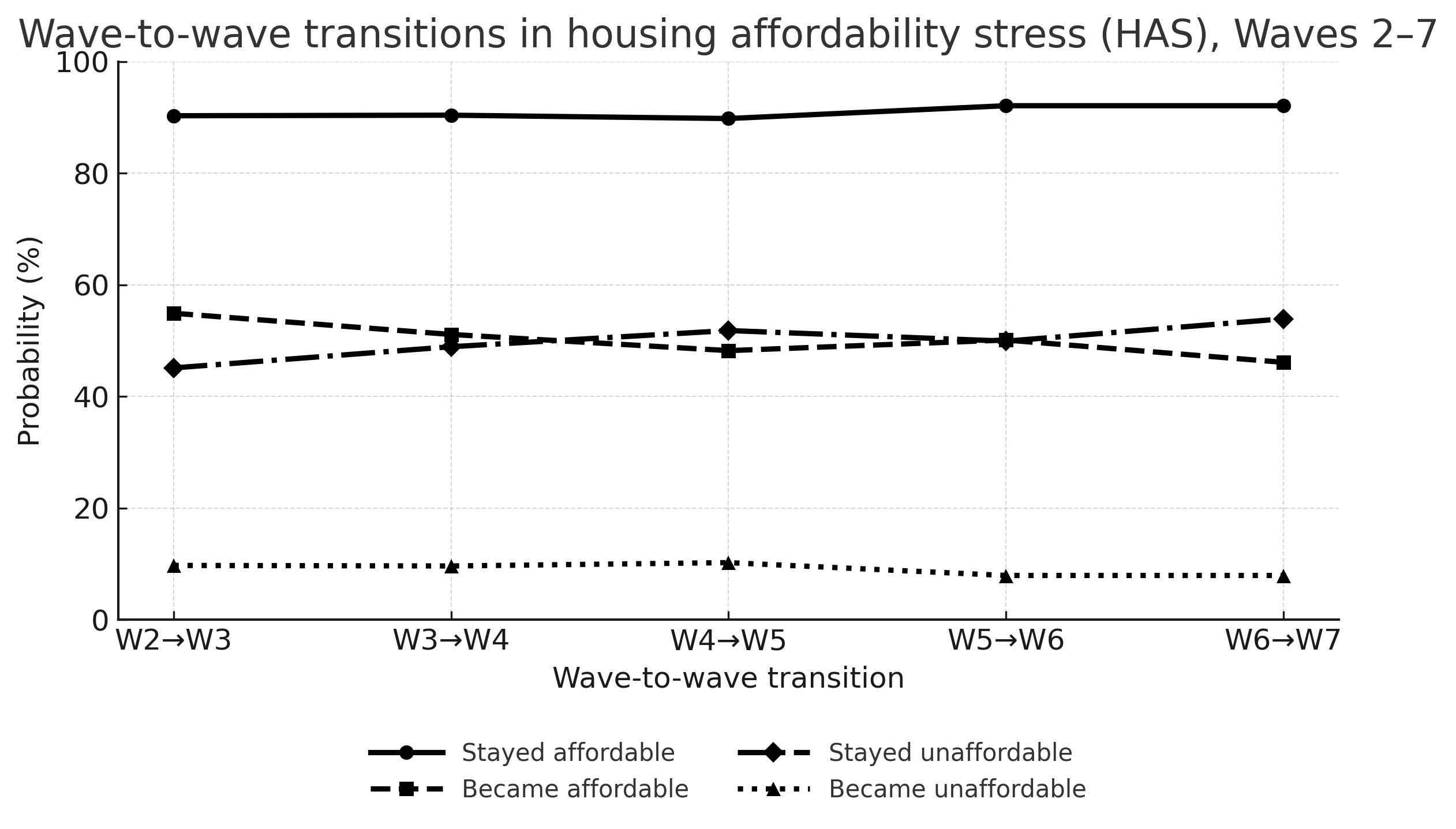


**Figure S2. Wave-to-wave transitions in housing affordability stress (HAS), Waves 2–7.**

Line plot showing probabilities of remaining or changing housing affordability status between consecutive survey waves. Percentages are based on children with non-missing HAS data at each wave (n = 3,773). Distinct line styles denote transition types: stayed affordable (solid), became affordable (dashed), stayed unaffordable (dash-dot), and became unaffordable (dotted).Abbreviation: HAS, housing affordability stress.
